# PTH Receptor Signaling in Osteocytes Governs Periosteal Bone Formation and Intracortical Remodeling

**DOI:** 10.1002/jbmr.304

**Published:** 2010-12-07

**Authors:** Yumie Rhee, Matthew R Allen, Keith Condon, Virginia Lezcano, Ana C Ronda, Carlo Galli, Naomi Olivos, Giovanni Passeri, Charles A O'Brien, Nicoletta Bivi, Lilian I Plotkin, Teresita Bellido

**Affiliations:** 1 Department of Anatomy and Cell Biology, Division of Endocrinology, Indiana University School of MedicineIndianapolis, IN, USA; 2 Department of Medicine, Division of Endocrinology, Center for Osteoporosis and Metabolic Bone Diseases, University of Arkansas for Medical SciencesLittle Rock, AR, USA; 3 Department of Medicine, Division of Endocrinology, Indiana University School of MedicineIndianapolis, IN, USA

**Keywords:** OSTEOCYTES, PTH RECEPTOR, PERIOSTEAL BONE FORMATION, WNT SIGNALING, INTRACORTICAL REMODELING

## Abstract

The periosteal and endocortical surfaces of cortical bone dictate the geometry and overall mechanical properties of bone. Yet the cellular and molecular mechanisms that regulate activity on these surfaces are far from being understood. Parathyroid hormone (PTH) has profound effects in cortical bone, stimulating periosteal expansion and at the same time accelerating intracortical bone remodeling. We report herein that transgenic mice expressing a constitutive active PTH receptor in osteocytes (DMP1-caPTHR1 mice) exhibit increased cortical bone area and an elevated rate of periosteal and endocortical bone formation. In addition, DMP1-caPTHR1 mice display a marked increase in intracortical remodeling and cortical porosity. Crossing DMP1-caPTHR1 mice with mice lacking the Wnt coreceptor, LDL-related receptor 5 (LRP5), or with mice overexpressing the Wnt antagonist *Sost* in osteocytes (DMP1-Sost mice) reduced or abolished, respectively, the increased cortical bone area, periosteal bone formation rate, and expression of osteoblast markers and Wnt target genes exhibited by the DMP1-caPTHR1 mice. In addition, DMP1-caPTHR1 lacking LRP5 or double transgenic DMP1-caPTHR1;DMP1-Sost mice exhibit exacerbated intracortical remodeling and increased osteoclast numbers, and markedly decreased expression of the RANK decoy receptor osteoprotegerin. Thus, whereas *Sost* downregulation and the consequent Wnt activation is required for the stimulatory effect of PTH receptor signaling on periosteal bone formation, the Wnt-independent increase in osteoclastogenesis induced by PTH receptor activation in osteocytes overrides the effect on *Sost*. These findings demonstrate that PTH receptor signaling influences cortical bone through actions on osteocytes and defines the role of Wnt signaling in PTH receptor action. © 2011 American Society for Bone and Mineral Research.

## Introduction

The skeleton is composed of cancellous or trabecular bone intertwined with bone marrow surrounded by a shell of cortical bone. Bone formation on the periosteal surface of cortical bone regulates the outer shape of bones and, in concert with activity on the endocortical surface, determines cortical thickness and bone size. Periosteal expansion significantly increases bone strength, independently of increases in areal bone mineral density.(1,2) Fractures in clinically relevant sites initiate in the cortical bone,(3) even in bones composed predominantly of cancellous bone such as the femoral neck.(4) Moreover, greater cortical bone mass may explain the higher resistance to vertebral fracture in males compared to females.(5,6) Despite the influence of cortical bone and specifically the periosteum on fracture prevention, the mechanisms that govern cortical bone biology and the response of this site to osteoporotic therapies are far from being understood.

Mechanical and hormonal stimuli influence periosteal bone formation during growth as well as in the adult skeleton.(7) In particular, parathyroid hormone (PTH) is a key stimulator of periosteal expansion.(8) Early studies in humans showed that asymptomatic patients with either primary hyperparathyroidism or hyperparathyroidism secondary to chronic kidney disease exhibit increased metacarpal outer diameter.(8) Consistent with this finding, patients with primary hyperparathyroidism exhibit 2- to 3-fold higher periosteal bone formation rate (BFR) than controls do.(9) Daily injections of PTH to osteoporotic patients also augment cortical bone width and increase BFR on both periosteal and endocortical surfaces.(10) In rodents and rabbits, intermittent PTH administration enhances periosteal bone formation(11–14) by a mechanism that might require insulin-like growth factor 1 signaling.(15,16) All this evidence notwithstanding, elevation of PTH does not always increase bone formation on the periosteal surface.(17–19) The bases for this apparent dichotomy are not understood.

In cancellous bone, PTH increases bone formation by mechanisms that seem to differ depending on the mode of elevation. The anabolic effect of intermittent PTH in rodents can be accounted for by prolonging the life span of mature osteoblasts in combination with the pro-differentiating effects of the hormone,(20–22) whereas chronic elevation of PTH increases osteoblast number by acting on osteocytes to suppress the expression of the bone formation inhibitor sclerostin, encoded by the *Sost* gene.(23,24) Recent evidence shows that transgenic mice expressing a constitutively active PTH receptor 1 exclusively in osteocytes (DMP1-caPTHR1 mice) exhibit reduced sclerostin expression, increased Wnt signaling, and increased cancellous bone volume.(25) In addition, these mice display elevated bone turnover markers. These findings suggest that previously unrecognized effects of PTH on osteocytes mediate at least some of the actions of the hormone on the skeleton. Whether PTH regulates cortical bone by acting on osteocytes was heretofore unknown.

We report here that osteocyte-specific constitutive activation of the PTH receptor leads to increased cortical bone area and an elevated rate of periosteal and endocortical bone formation. In addition, DMP1-caPTHR1 mice display a remarkable increase in intracortical remodeling associated with increased cortical porosity. Removal of the Wnt coreceptor, LDL-related receptor 5 (LRP5), or osteocyte-targeted overexpression of *Sost*, whose product sclerostin binds to both LRP5 and 6, reduced or abolished, respectively, the increased cortical bone area, periosteal BFR, and expression of osteoblast markers and Wnt target genes exhibited by the DMP1-caPTHR1 mice. In addition, interference with the Wnt pathway exacerbated intracortical remodeling and the increase in osteoclast number displayed by the DMP1-caPTHR1 mice and markedly decreased the expression of the RANK decoy receptor osteoprotegerin (OPG). These findings show that PTH receptor signaling influences cortical bone through actions on osteocytes and defines the role of Wnt signaling in PTH receptor action.

## Materials and Methods

### Generation of DMP1-caPTHR1 and DMP-Sost transgenic mice and crosses with LRP5^−/−^ mice or DMP1-Sost mice

Generation of DMP1-caPTHR1 transgenic mice has been described previously.(25) DMP1-Sost transgenic mice were generated by inserting the human *Sost* cDNA (I.M.A.G.E. clone ID 40009482, American Tissue Culture Collection, Manassas, VA, USA) downstream from a 12-kb DNA fragment containing 8 kb of the 5′-flanking region, the first exon, the first intron, and 17 bp of exon 2 of the murine DMP1 gene(26) and upstream from a 140-bp fragment containing the rabbit beta-globin polyadenylation sequence. Transgenic mice were produced by microinjection of purified DNA into pronuclei of C57BL/6 mice at the transgenic mouse core facility of the University of Arkansas for Medical Sciences. DMP1-Sost mice were born at the expected Mendelian frequency, were fertile, and exhibited normal size and weight. DMP1-caPTHR1 and DMP1-Sost mouse colonies were maintained by breeding mice hemizygous for the transgene with wild type C57BL/6 mice. All transgenic mice used in these studies were hemizygous. The DMP1-caPTHR1 and DMP1-Sost transgenes were detected in bone but not in skeletal muscle, heart, brain, kidney, duodenum, or colon(25) (see also Supplemental Fig. 1*A*, *B*).

DMP1-caPTHR1 mice were crossed with mice lacking LRP5(27) or with DMP1-Sost mice to obtain mice expressing the DMP1-caPTHR1 transgene in an LRP5-deficient background or mice expressing both the DMP1-caPTHR1 and the DMP1-Sost transgenes. Animals were fed a regular diet (Harlan/Teklad 7001, Indianapolis, IN, USA) and water ad libitum and were maintained on a 12-hour light/dark cycle. Animal protocols were approved by the Institutional Animal Care and Use Committees of the University of Arkansas for Medical Sciences and the Indiana University School of Medicine.

### Analysis of skeletal phenotypes

Analysis of skeletal phenotypes was performed in mice of 4.6 and 12 weeks of age as indicated in the figure legends. Bone mineral density (BMD) was determined by dual energy x-ray absorptiometry (DXA) using a PIXImus II densitometer (GE Medical Systems, Madison, WI, USA) as previously described.(25) Mice were anesthetized by inhalation of 2.5% isoflurane (Abbott Laboratories, Abbott Park, IL, USA) mixed with O_2_ (1.5 liters/minute). BMD measurements of the total body excluding the head, lumbar spine, and femur were taken. For micro-CT analysis, femora and L3 vertebra were dissected, cleaned of soft tissue, stored in 70% ethanol, and scanned at 6-micron resolution (Skyscan 1172, SkyScan, Kontich, Belgium). For histomorphometric analysis, femora and calvariae were dissected, fixed, and embedded in methyl methacrylate. Fluorochrome labeling of the bones was performed by intraperitoneal injections of calcein (30 mg/kg) and alizarin (50 mg/kg; Sigma Chemical, St. Louis, MO, USA) administered 7 and 2 days, respectively, before the mice were killed, as previously described.(25) Thick cross-sections of undecalcified femora at the mid-diaphysis were prepared using a diamond-embedded wire saw (Histosaw, Delaware Diamond Knives, Wilmington, DE, USA) and ground to a final thickness of 30–35 µm. Frontal-plane 8 µm–thick calvarial sections were obtained 2 mm anterior to the junction between the frontoparietal and sagittal sutures using an Automated Rotary Microtome Leica RM2255 (Leica Microsystems, Bannockburn, IL, USA). Sections were viewed at 20–40x magnification on a Leitz DMRXE microscope (Leica Mikroskopie und System GmbH, Wetzlar, Germany). Images were captured using a SPOT digital camera (Diagnostic Instruments, Sterling Heights, MI, USA). Single- and double-labeled perimeter and interlabel width were measured on the periosteal and endocortical surfaces of 2 femoral sections per mouse and on the outer and inner periosteal surfaces of 1 calvarial section per mouse, using a semiautomatic analysis system (Bioquant OSTEO 7.20.10, Bioquant Image Analysis Co., Nashville, TN, USA) attached to a microscope equipped with an ultraviolet light source (Optiphot 2 microscope, Nikon, Melville, NY, USA). Von Kossa was used to stain mineralized bone, followed by enzyme histochemistry for TRAPase and counterstain with Gill's III hematoxylin for visualizing osteoclasts in calvarial sections.(25,28) TRAPase-positive multinucleated cells were counted using the OsteoMeasure High Resolution Digital Video System (OsteoMetrics, Decatur, GA, USA) attached to an Olympus BX51TRF microscope (Olympus America, Center Valley, PA, USA). Osteoclast number was expressed per bone area. The terminology and units used are those recommended by the Histomorphometry Nomenclature Committee of the American Society for Bone and Mineral Research.(29)

### Bone turnover markers

Plasma osteocalcin and C-telopeptide fragments of type I collagen (CTX) were measured using enzyme-linked immunosorbent assays (Biomedical Technologies, Stoughton, MA, USA, and Immunodiagnostic Systems, Fountain Hill, AZ, USA, respectively), as published.(25)

### Immunohistochemistry

Detection of sclerostin expression on paraffin-embedded tibiae from 6-week-old mice was performed as previously described.(24,25) Briefly, sections were deparaffinized, treated with 3% H_2_O_2_ to inhibit endogenous peroxidase activity, blocked with rabbit or goat serum, and then incubated with a 1:100 dilution of goat polyclonal antimouse sclerostin antibody (R&D Systems, Minneapolis, MN, USA) or rabbit polyclonal anti-human sclerostin antibody (Abcam, Cambridge, MA, USA), respectively. Sections were then incubated with rabbit anti-goat horseradish peroxidase-conjugated secondary antibody (Santa Cruz Biotechnologies, Santa Cruz, CA, USA) or goat antirabbit biotinylated secondary antibody followed by avidin-conjugated peroxidase (Vectastain Elite ABC Kit; Vectora Laboratories, Burlingame, CA, USA). Color was developed with a diaminobenzidine substrate chromogen system (Dako, Carpinteria, CA, USA). Nonimmune IgGs were used as negative controls.

### Quantitative PCR

Whole tibiae from 12-week-old DMP1-caPTHR1;LRP5^−/−^ mice or ulnae from 6-week-old DMP1-caPTHR1;DMP1-Sost mice were snap frozen. Total RNA was purified using Ultraspec reagent (Biotecx Laboratories, Houston, TX, USA) according to the manufacturer's instructions. Gene expression was analyzed by quantitative PCR as previously described(25) using primer probe sets from Applied Biosystems (Foster City, CA, USA) or from Roche Applied Science (Indianapolis, IN, USA). Relative mRNA expression levels were normalized to the housekeeping gene ribosomal protein S2 using the ΔCt method.

### Statistical analysis

Data were analyzed using SigmaStat (SPSS Science, Chicago, IL, USA). All values are reported as the mean ± standard deviations (SD). Differences between group means were evaluated using Student's *t*-test or two-way ANOVA.

## Results

### Mice expressing a constitutively active PTHR1 in osteocytes exhibit increased cortical bone area and elevated periosteal and endocortical bone formation

**Fig. 1:**
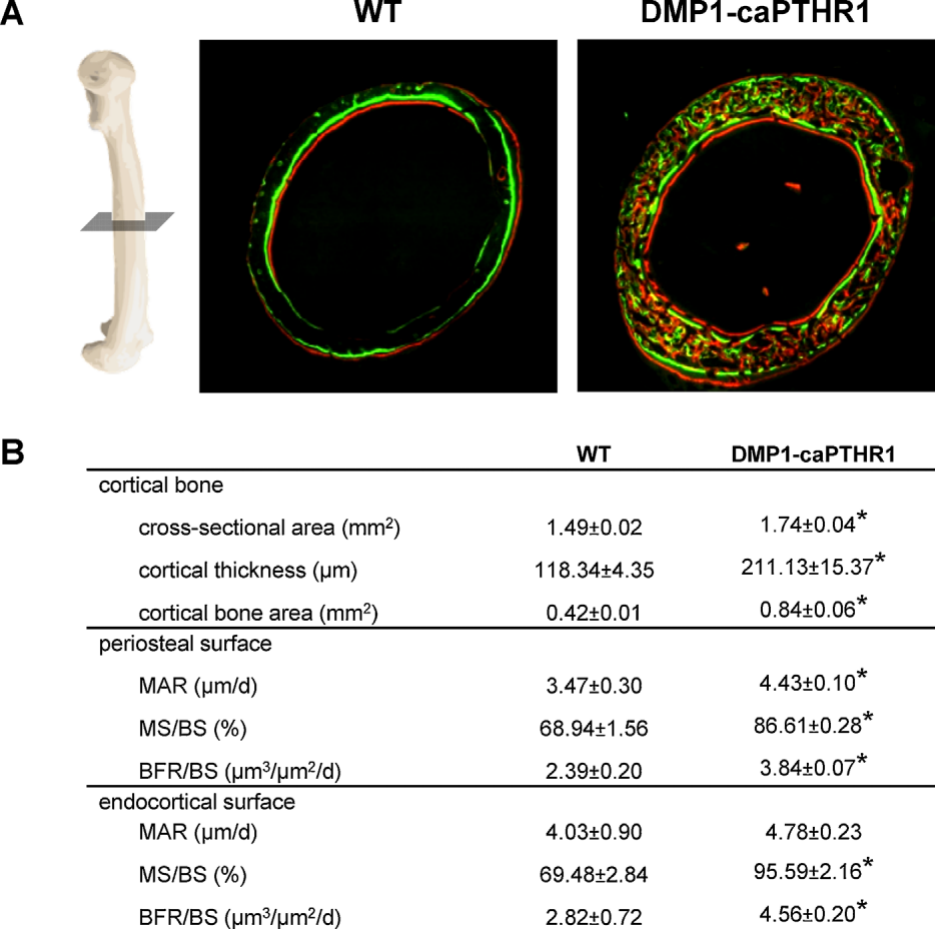
Activation of PTHR1 signaling in osteocytes increases periosteal and endocortical bone formation. *(A)* Representative images of histologic sections showing calcein and alizarin double labeling in femoral mid-diaphyses from 4.6-week-old DMP1-caPTHR1 mice and WT littermates. *(B)* Static and dynamic histomorphometric measurements were performed in 3 male mice per group. Values are means ± SD. *p < .05 versus WT mice.

Microscopic examination of cross-sectional cuts of the femur at mid-diaphysis of 4.6-week-old mice showed larger bone diameter in DMP1-caPTHR1 bones (Fig. 1*A*). Histomorphometric analysis showed that the total cross-sectional area was 17% higher and cortical thickness and cortical bone area approximately double in the DMP1-caPTHR1 mice (Fig. 1*B*). These geometry differences resulted from enhanced activity on both cortical surfaces. Also, whereas wild type (WT) littermate midshafts exhibited the classical pattern of modeling with fluorochrome labeling present only in part of the periosteal and the opposite endocortical surfaces, fluorochrome incorporation in DMP1-caPTHR1 bones was observed along the entire periosteal and endocortical surface (Fig. 1*A*). Mineralizing surface per bone surface (MS/BS) was markedly increased in the periosteal and endocortical surface of DMP1-caPTHR1 mid-diaphyses relative to those of WT littermates. Mineral apposition rate (MAR) was also increased in both periosteal and endocortical surfaces, although the effect was statistically significant only for the periosteum (Fig. 1*B*). This resulted in a significant increase in periosteal and endocortical BFR on both surfaces in DMP1-caPTHR1 mice. Similar changes were found in male and female mice.

**Fig. 2:**
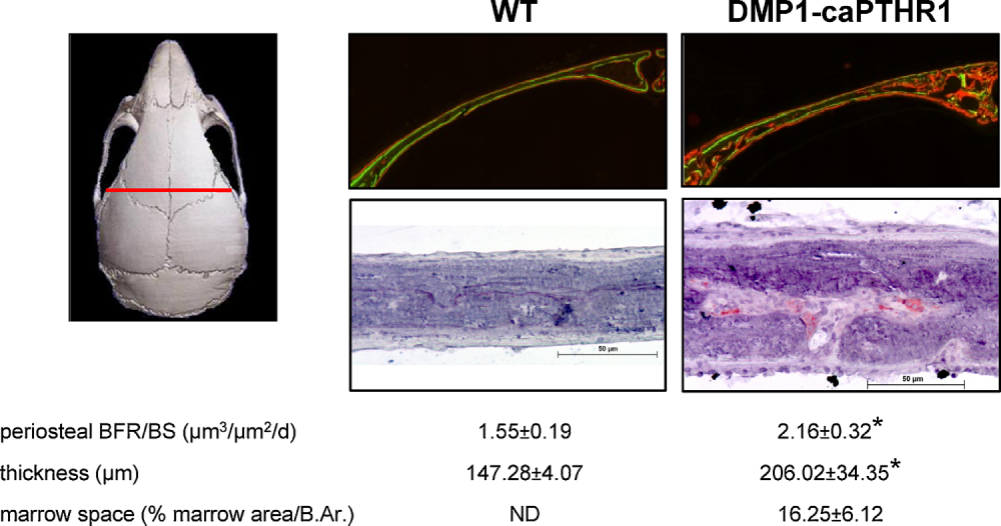
Activation of PTHR1 signaling in osteocytes increases periosteal bone formation in calvaria and induces intracortical remodeling. Representative images of histologic sections showing calcein and alizarin double labeling in transverse sections taken 2 mm anterior to the junction between the frontoparietal and sagittal sutures (indicated by the red line) from 4.6-week-old WT and DMP1-caPTHR1 mice (*top*). TRAPase staining of calvarial sections shows abundant osteoclasts in bones from DMP1-caPTHR1 mice (*bottom*). Bar indicates 50 µm. Histomorphometric analysis was performed in the mid-third of one frontal bone, excluding the area around the sutures. Periosteal BFR is the average of values of outer and inner periosteum. Calvarial marrow space was calculated by measuring histomorphometrically the area occupied by marrow versus bone in the mid-third of one frontal bone. Values are means ± SD; *n* = 3 male mice per group. *p < .05 versus WT mice. ND = not detected.

We next examined the periosteal surface in the compact bone of the skull, which is formed by intramembranous ossification (Fig. 2). BFR measured on both the outer and inner calvarial periosteal surface was also higher in the DMP1-caPTHR1 mice. Calvarial thickness measured in transverse sections was 1.4-fold higher in DMP1-caPTHR1 mice.

### DMP1-caPTHR1 mice exhibit increased bone remodeling in cortical bone

Previous histomorphometric analysis of cancellous bone at the distal femur of DMP1-caPTHR1 indicated that not only osteoblast but also osteoclast perimeter was elevated and quiescent surface was decreased.(25) We also found abundant fluorochrome incorporation, an index of intracortical (endosteal) bone formation, in cortical bone at the femoral diaphysis (Fig. 1*A*) as well as in calvaria (Fig. 2) of DMP1-caPTHR1 mice. In addition, calvarial porosity measured histomorphometrically as percent marrow area per bone area was observed only in calvariae of the DMP1-caPTHR1 mice. In addition, abundant osteoclasts were present only in DMP1-caPTHR1 calvaria. Taken together with the previous findings that plasma and urine markers of bone resorption are elevated in these mice,(25) these results indicate that activation of PTH receptor signaling in osteocytes leads to increased bone remodeling in cortical as well as cancellous bone.

### The periosteal bone phenotype of the DMP1-caPTHR1 mice is partially reversed in the absence of LRP5 and abolished by *Sost* overexpression

**Fig. 3:**
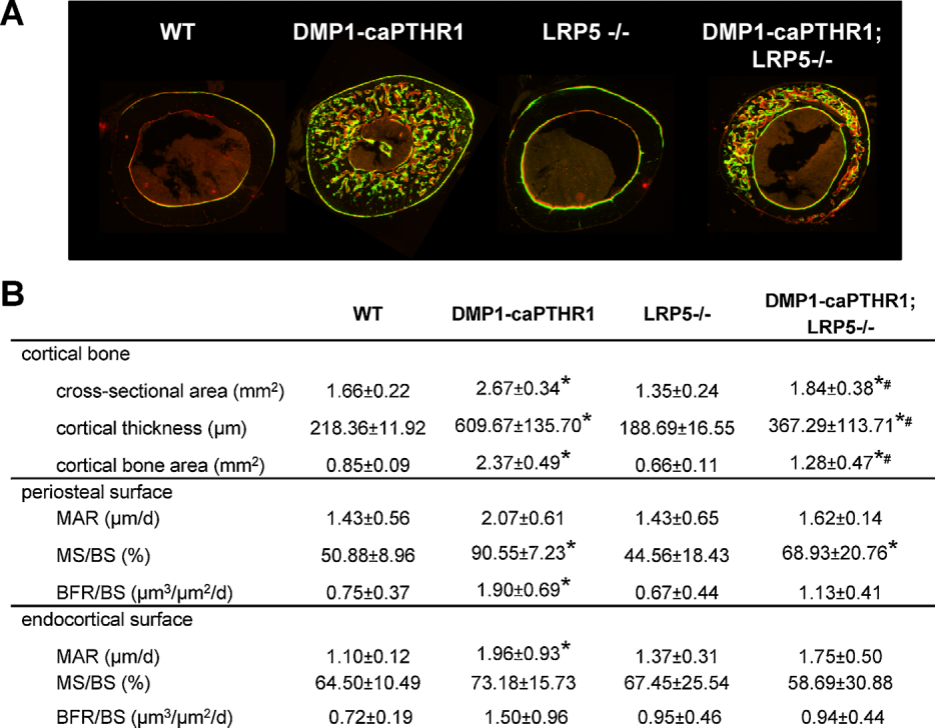
Periosteal bone formation induced by PTH receptor signaling in osteocytes is reduced in mice lacking LRP5. *(A)* Representative images of histologic sections show fluorochrome incorporation in femoral mid-diaphyses of 12-week-old WT and DMP1-caPTHR1 mice with or without LRP5. *(B)* Static and dynamic histomorphometric measurements were determined in 3–4 male and female mice per group. Values are means ± SD. *p < .05 versus respective controls without the DMP1-caPTHR1 transgene; ^#^*p* < .05 versus DMP1-caPTHR1 mice.

Previous findings had shown that the increased cancellous bone of the DMP1-caPTHR1 mice was reduced in mice also lacking the Wnt coreceptor LRP5.(25) To examine whether a similar mechanism was involved in the effect of the DMP1-caPTHR1 transgene on cortical bone, we analyzed the femoral mid-diaphysis of the DMP1-caPTHR1 mice crossed with LRP5^−/−^ mice. As shown for younger mice in Fig. 1, 3-month-old DMP1-caPTHR1 mice exhibited increased cross-sectional area, cortical thickness, and cortical bone area (Fig. 3*A, B*). The increase in these parameters was significantly reduced, but not eliminated, in DMP1-caPTHR1 mice lacking LRP5. Similarly, the higher periosteal BFR and mineralizing surface observed even in these 3-month-old DMP1-caPTHR1 mice was partially reversed in the absence of LRP5. At this age, however, the effect of the DMP1-caPTHR1 transgene on bone formation on the endocortical surface did not reach significance.

**Fig. 4:**
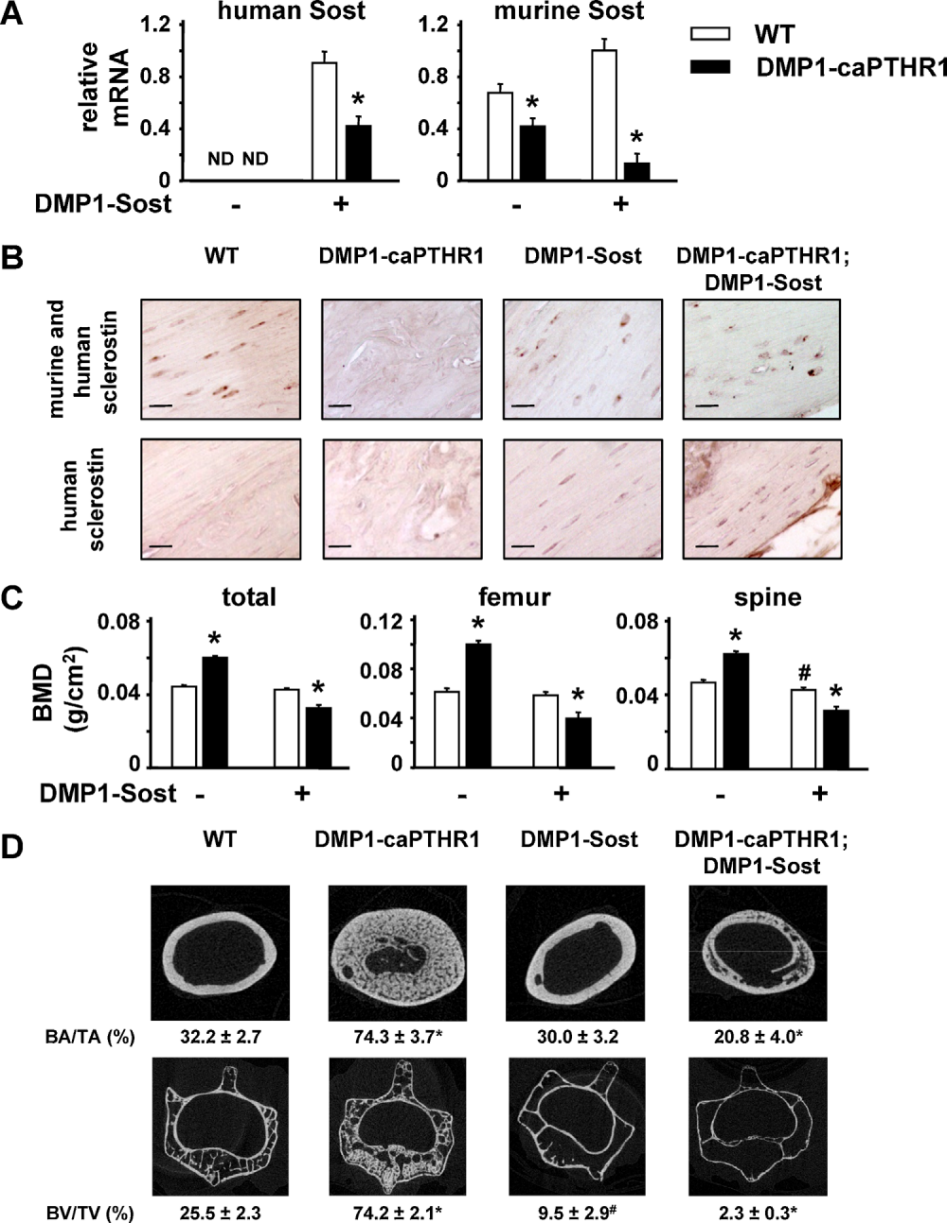
The high bone mass phenotype of the DMP1-caPTHR1 mice is abolished by overexpression of *Sost* in osteocytes. *(A)* Expression of human or murine *Sost* was detected in ulnae lysates by quantitative PCR; *n* = 3–5 female mice per group. ND, not detected. *(B)* Sclerostin expression was detected by immunohistochemistry in tibiae using an antimurine sclerostin antibody that also recognizes human sclerostin or using a specific antihuman sclerostin antibody. Bar indicates 10 µm. *(C)* Total, femoral, and spinal BMD measured by DXA in 8-week-old mice expressing the DMP1-caPTHR1 and/or the DMP1-Sost transgenes and in WT littermates is shown; *n* = 18–32 male and female mice per group. *(D)* Representative micro-CT images of cross-sections of femoral mid-diaphyses (*upper panel*) and L3 (*lower panel*) of WT and DMP1-caPTHR1 mice with or without the DMP1-Sost transgene. Cortical bone area as a percentage of total area within the periosteal circumference (BA/TA) from the femoral diaphysis and trabecular BV/TV of the L3 body were measured in 4–7 male and female mice per group. Values represent the mean ± SD. *p < .05 versus respective controls without the DMP1-caPTHR1 transgene; ^#^*p* < .05 versus WT mice.

The persistent effect of the DMP1-caPTHR1 transgene on cortical bone in LRP5-deficient mice might result from remaining increased Wnt signaling through LRP6. Sclerostin interacts with and inhibits signaling through both LRP5 and LRP6.(30) To directly address the role of suppressed *Sost*/sclerostin expression in the actions of the caPTHR1, we generated mice overexpressing the human *Sost* gene in osteocytes (DMP1-Sost mice) and crossed them with DMP1-caPTHR1 mice. Expression of human *Sost* mRNA was found only in DMP1-Sost mice, whereas endogenous murine *Sost* was detected at similar levels in mice expressing or not expressing the DMP1-Sost transgene (Fig. 4*A*). Mice expressing the DMP1-caPTHR1 transgene exhibited decreased expression of endogenous murine *Sost*, regardless of whether the DMP1-Sost transgene was expressed. Immunohistochemistry using an antibody against murine sclerostin that also recognizes the human protein demonstrated sclerostin expression in osteocytes in bone sections of WT and DMP1-Sost mice (Fig. 4*B*). Consistent with previous findings,(25) sclerostin expression was markedly decreased in osteocytes of DMP1-caPTHR1 mice. On the other hand, DMP1-caPTHR1 mice co-expressing the DMP1-Sost transgene exhibited persistent high expression of sclerostin (Fig. 4*B*, *upper panel*). Although mRNA for the human *Sost* transgene was expressed at lower levels in animals co-expressing the DMP1-caPTHR1 transgene (Fig. 4*A*, *left graph*), human sclerostin detected using an antibody specific for human protein was present at apparent similar levels in DMP1-Sost mice and DMP1-Sost;DMP1-caPTHR1 mice (Fig. 4*B*, *lower panel*).

BMD measurements taken at 4 weeks, 6 weeks (not shown), and 8 weeks (Fig. 4*C*) of age revealed no significant change in total or femoral BMD in DMP1-Sost mice. However, spinal BMD was significantly lower at all ages (Fig. 4*C* and not shown). Micro-CT analysis showed no changes in cortical bone area in the femoral mid-diaphyses but a dramatic decrease in cancellous bone volume in the vertebrae of DMP1-Sost mice (Fig. 4*D*). Despite this apparent differential effect of the DMP1-Sost transgene on cortical and cancellous bone, the increase in BMD exhibited by DMP1-caPTHR1 mice was completely abolished at both the femur and spine in mice expressing both DMP1-caPTHR1 and the DMP1-Sost transgenes (Fig. 4*C, D*). Furthermore, the double transgenic mice exhibited significantly lower BMD in all sites than mice expressing solely the DMP1-Sost transgene, because of increased osteoclast activity, as will be discussed.

**Fig. 5:**
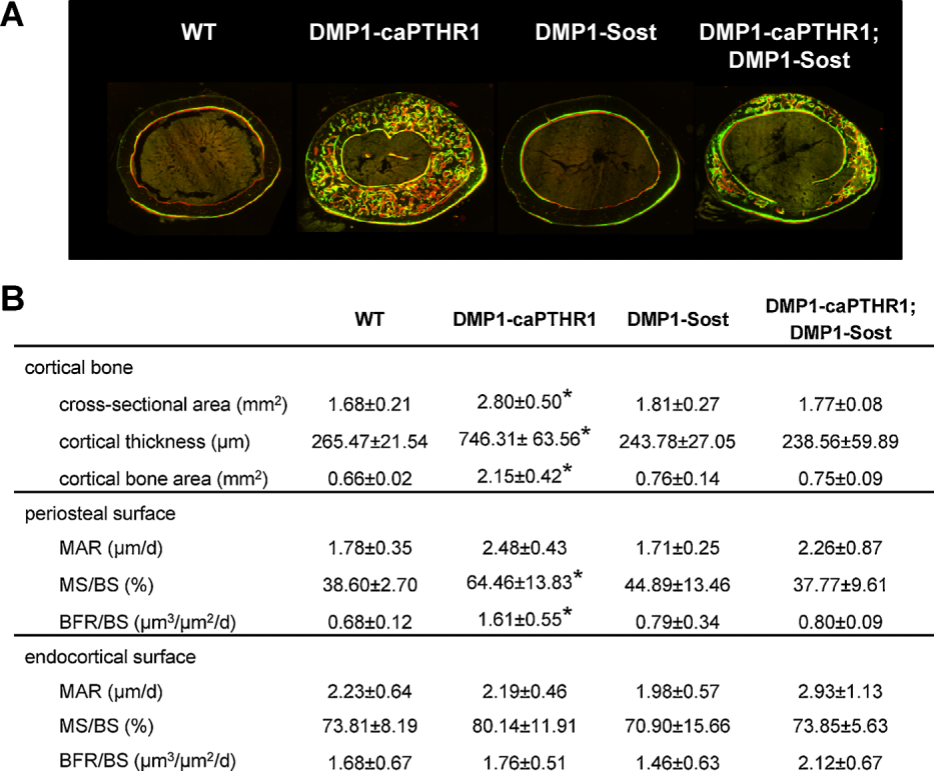
Periosteal bone formation induced by PTH receptor signaling in osteocytes is abolished by overexpression of *Sost* in osteocytes. *(A)* Representative images of histologic sections show fluorochrome incorporation in femoral mid-diaphyses of 10.5-week-old WT and DMP1-caPTHR1 mice with or without the DMP1-Sost transgene. *(B)* Static and dynamic histomorphometric measurements were determined in 3–5 mice per group. Values are means ± SD. *p < .05 versus WT mice.

In contrast to the incomplete effect of deleting LRP5, the higher cortical bone area exhibited by the DMP1-caPTHR1 mice was completely eliminated in mice also expressing the DMP1-Sost transgene (Fig. 5). Thus, total cross-sectional area, cortical thickness, cortical bone area, and periosteal BFR in the double transgenic mice were similar to those observed in DMP1-Sost mice or in nontransgenic mice. *Sost* overexpression also reduced the increased thickness and periosteal bone formation induced by the DMP1-caPTHR1 transgene in calvaria (Supplemental Fig. 2).

### The elevated intracortical bone remodeling exhibited by the DMP1-caPTHR1 mice is still present in the absence of LRP5 and it is exacerbated by *Sost* over-expression

**Fig. 6:**
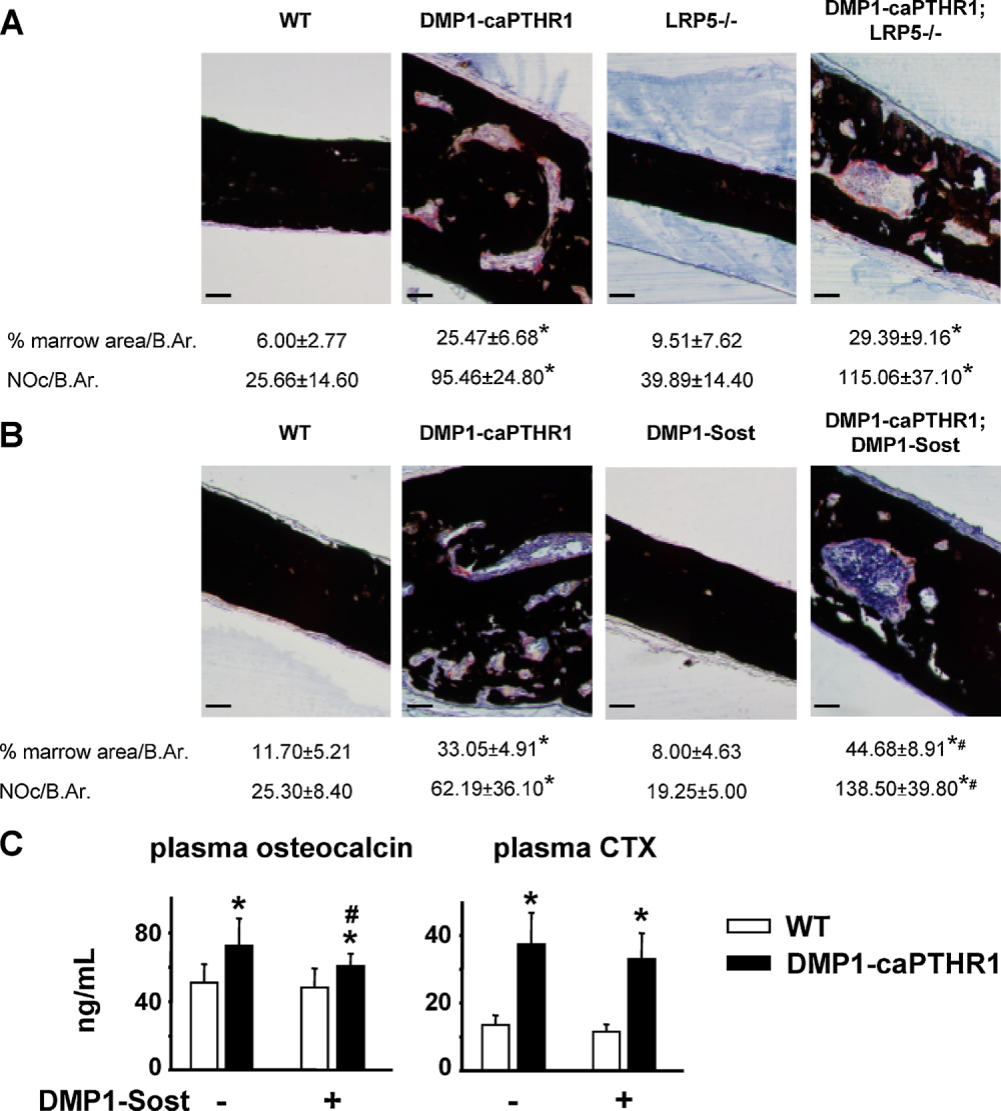
The increased bone resorption induced by PTH receptor signaling in osteocytes persists in the absence of LRP5 and is exacerbated by *Sost* overexpression. *(A,B)* TRAPase/von Kossa staining of calvarial sections and histomorphometric quantification of calvarial marrow space, expressed as the area occupied by marrow versus bone in the mid-third of one frontal bone, and of osteoclasts, expressed as number per bone area. *n* = 4 mice/group. Bar indicates 50 µm. *(C)* Osteocalcin and CTX were measured in plasma of 10.5-week-old DMP1-caPTHR1 mice, with and without the DMP1-Sost transgene. Bars represent the mean ± SD; *n* = 7–23 mice per group. *p < .05 versus WT and LRP5^−/−^ or DMP1-Sost mice, respectively; ^#^*p* < .05 versus DMP1-caPTHR1 mice.

Despite the lower periosteal bone formation induced by the DMP1-caPTHR1 transgene in LRP5^−/−^ mice or in mice overexpressing *Sost* in osteocytes, DMP1-caPTHR1;LRP5^−/−^ and DMP1-caPTHR1;DMP1-Sost mice displayed persistently higher intracortical remodeling resembling that in DMP1-caPTHR1 mice (Fig. 6). Thus, abundant intracortical fluorochrome incorporation was still observed in sections of femoral mid-diaphysis and calvaria (Figs. 3, S2, and 5). Calvarial marrow space was similarly higher in DMP1-caPTHR1 mice lacking or expressing LRP5 (Fig. 6*A*), and double transgenic DMP1-caPTHR1;DMP1-Sost mice exhibited even higher calvarial marrow space than mice expressing only the DMP1-caPTHR1 transgene (Fig. 6*B*). Osteoclast number per bone area quantified in von Kossa/TRAPase calvarial sections displayed a similar profile (Fig. 6*A, B*). Circulating levels of both formation (osteocalcin) and resorption (CTX) markers were increased in DMP1-caPTHR1 mice. CTX was still elevated in DMP1-caPTHR1;DMP1-Sost mice (Fig. 6*C*). However, osteocalcin levels in DMP1-caPTHR1;DMP1-Sost mice were significantly lower than in DMP1-caPTHR1 mice, as previously shown for DMP1-caPTHR1;LRP5^−/−^ mice.(25)

### Interference with the Wnt pathway decreased the expression of Wnt target genes and osteoblast markers induced by PTH receptor activation in osteocytes, but osteoclastogenic cytokines and osteoclast markers remained increased

**Fig. 7:**
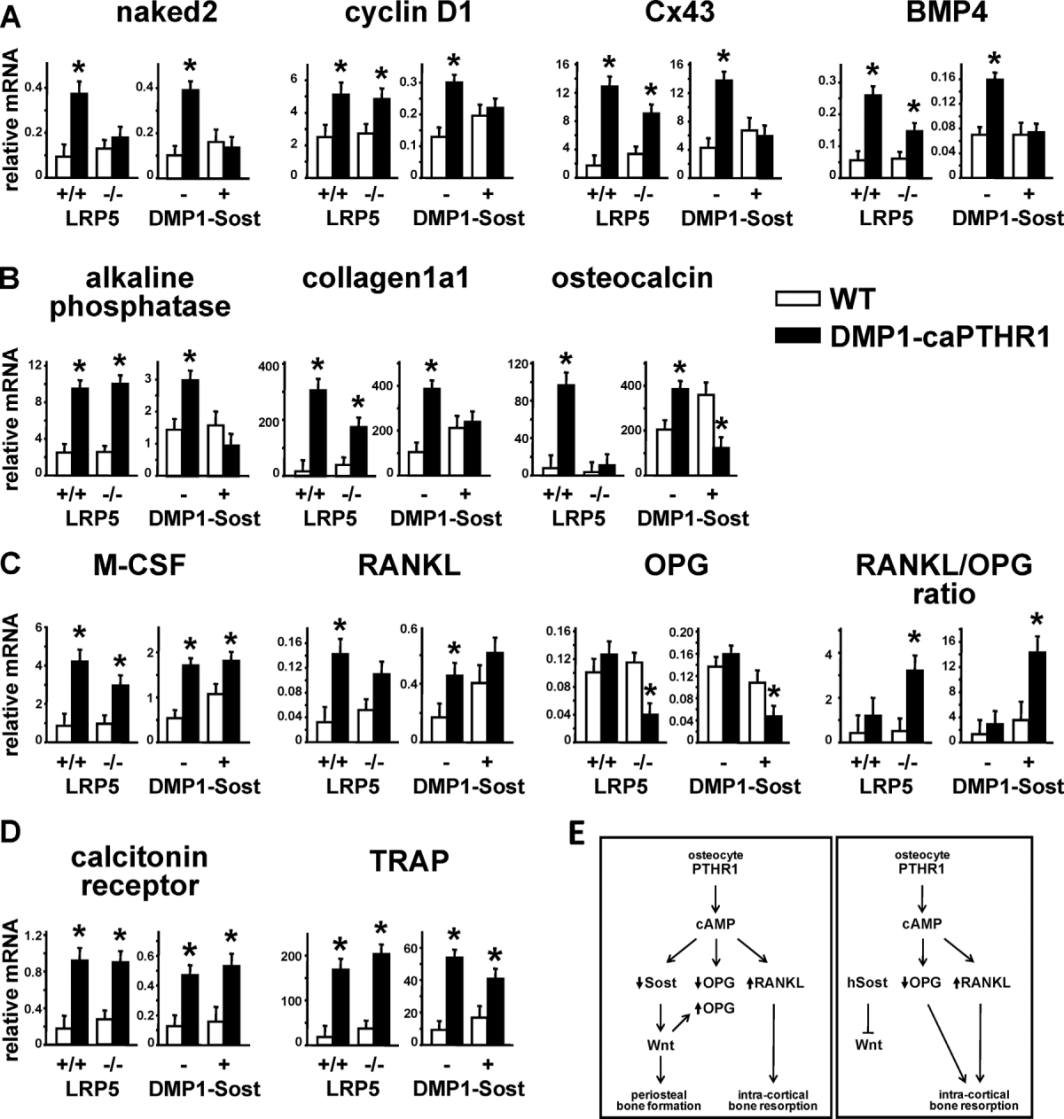
The increased expression of Wnt target genes and osteoblast markers induced by PTH receptor activation in osteocytes was obliterated by interfering with the Wnt pathway, but osteoclast markers were still elevated and OPG was markedly reduced. *(A–D)* Gene expression was measured by quantitative PCR. Results are expressed relative to the housekeeping gene ribosomal protein S2. Bars represent mean ± SD of 4–5 mice per group. *p < .05 versus respective controls without the DMP1-caPTHR1 transgene. *(E)* Schematic representation of the effects on cortical bone of PTH receptor signaling in osteocytes. Constitutive activation of PTH receptor in osteocytes (DMP1-caPTHR1 mice) leads to cAMP-dependent *Sost* downregulation and increased Wnt signaling, which in turn stimulates periosteal bone formation. PTH receptor activation in osteocytes also increases RANKL expression and intracortical remodeling. However, OPG expression is not affected, likely resulting from opposing effects on the expression of the gene by cAMP (reduction) and Wnts (elevation). When PTH receptor activation is combined with reduced Wnt signaling (LRP5 deficiency or *Sost* overexpression), the cAMP-mediated decrease in OPG prevails, leading to a markedly increased RANKL/OPG ratio and exacerbated bone resorption in the cortex.

Long bones from DMP1-caPTHR1 mice exhibited increased expression of Wnt target genes and of osteoblast and osteoclast markers (Fig. 7). Gene expression was remarkably affected in DMP1-caPTHR1 mice crossed with LRP5 null mice or with DMP1-Sost mice. Thus, the higher expression of the recognized Wnt target genes naked2, cyclin D1, Cx43, and BMP4(25,31) observed in the DMP1-caPTHR1 mice was eliminated in mice also expressing the Sost transgene (Fig. 7*A*). Naked2 expression was also decreased to WT values by deletion of LRP5. However, expression of cyclin D1 was barely affected, whereas Cx43 and BMP4 expression was reduced, although not eliminated, in DMP1-caPTHR1;LRP5^−/−^ mice. Consistent with the higher periosteal bone formation, the osteoblast markers alkaline phosphatase, collagen 1a1, and osteocalcin were elevated in DMP1-caPTHR1 mice (Fig. 7*B*). Deletion of LPR5 did not affect alkaline phosphatase expression, decreased collagen 1a1 expression only partially, and completely blocked osteocalcin expression. *Sost* overexpression abolished the increases in all these genes exhibited by the DMP1-caPTHR1 mice.

In contrast, and consistent with the persistently increased bone remodeling exhibited by DMP1-caPTHR1 mice crossed with LRP5^−/−^ or with DMP1-Sost mice, the osteoclast-specific genes calcitonin receptor and TRAP remained elevated in DMP1-caPTHR1;LRP5^−/−^ mice or in DMP1-caPTHR1;DMP1-Sost mice (Fig. 7*D*). Expression of the osteoclastogenic cytokines M-CSF and RANKL was increased in DMP1-caPTHR1 mice, and their levels remained elevated, although not significantly for RANKL, in DMP1-caPTHR1 mice crossed with LRP5^−/−^ or DMP1-Sost mice (Fig. 7*C*). OPG expression, which was not different in DMP1-caPTHR1 mice compared to WT littermates (Fig. 7*C*), was lower in DMP1-caPTHR1;LRP5^−/−^ mice or in DMP1-caPTHR1;DMP1-Sost mice. This resulted in a higher RANKL/OPG ratio in animals expressing the DMP1-caPTHR transgene and lacking LRP5 or overexpressing *Sost*.

## Discussion

Early studies showing localization of radiolabeled PTH in osteocytes and morphological changes in these cells upon hormonal treatment had suggested the regulation of osteocyte function by PTH.(32,33) The findings that PTH decreases the expression of the osteocyte-derived inhibitor of bone formation sclerostin suggested a mechanism by which the hormone could increase bone mass through actions on osteocytes.(23,24) More recently, we have shown that expression of a constitutively active mutant of the PTH receptor in osteocytes is sufficient to increase mass and remodeling in cancellous bone. (25) In the present report, we found that DMP1-caPTHR1 mice exhibit accelerated bone formation on the periosteal and endocortical surfaces and increased intracortical remodeling, thereby showing that PTH receptor signaling in osteocytes governs periosteal bone formation and turnover also in cortical bone (Fig. 7*E*). These actions of PTH receptor activation were observed in bones formed by either endochondral or intramembranous ossification. Moreover, higher periosteal apposition was found in both male and female mice regardless of the age of the animals, showing that the effect of PTH receptor activation in osteocytes overrides the recognized action of growth as well as androgens on periosteal bone formation and size of long bones.(34)

The current findings also show that the anabolic effect of PTH receptor signaling on the periosteal surface of cortical bone is dependent on inhibition of sclerostin expression, as the effect of the caPTHR1 transgene was abolished by overexpressing *Sost* in osteocytes. However, as judged by the persistent fluorochrome incorporation, bone formation coupled to the intracortical resorption exhibited by the DMP1-caPTHR1 mice was not decreased by removing LRP5 or overexpressing *Sost*. These findings suggest that sclerostin differentially regulates modeling-based periosteal bone formation versus remodeling-based endosteal bone formation. Further studies are warranted to specifically address the role of resorption on bone formation induced by PTH receptor signaling in osteocytes.

Expression of the endogenous (murine) *Sost* gene was lower in DMP1-caPTHR1 mice, regardless of whether the DMP1- (human) Sost transgene was co-expressed. This is consistent with earlier findings that PTH downregulates the expression of *Sost*/sclerostin in vivo and in vitro(23,24) and with our previous report.(25) Keller and colleagues showed that inhibition of *Sost* expression by PTH is exerted through modulation of the transcription factor Mef2c through binding on the same distant regulatory region of the *Sost* gene promoter that confers bone-specific expression of the gene.(35) Expression of the human Sost transgene in our DMP1-Sost mice is directed by the DMP1 promoter, not by the regulatory regions of the *Sost* gene. Therefore, the lower levels of human *Sost* mRNA observed in animals expressing both the DMP1-Sost and the DMP1-caPTHR1 transgenes might result not from direct gene regulation by PTHR activation but rather from changes either in osteocyte number or in their state of maturation. Nevertheless, even when human *Sost* mRNA expression was lower, human sclerostin protein was still detected at high levels in the double transgenic mice.

The mechanism by which sclerostin, the product of the *Sost* gene secreted by osteocytes, inhibits bone formation is not completely understood. However, the current knowledge indicates that sclerostin binds with high affinity to LRP6 and LRP5, transmembrane proteins that together with frizzled receptors mediate the actions of Wnt ligands. It is believed that sclerostin binding interferes with signaling downstream of these receptors, thereby antagonizing the pro-differentiating and survival actions of Wnts on cells of the osteoblastic lineage.(36,37) It was recently shown that sclerostin also associates with LRP4, another member of the LRP family of proteins,(38,39) although its role in Wnt signaling is still uncertain. Nevertheless, it is possible that the remaining phenotypic features of PTH receptor activation in the absence of LRP5 observed in the current study in cortical bone, as well as in cancellous bone in our previous study,(25) result from Wnt signaling through alternative LRPs.

Because the increase in bone mass exhibited by DMP1-caPTHR1 mice is reduced in mice lacking LRP5, and because a recent report indicated that LRP5 increases bone formation by inhibiting serotonin synthesis in the duodenum,(40) we examined whether the high bone mass of the DMP1-caPTHR1 mice was associated with decreased intestinal expression of Tph1, the enzyme that controls serotonin synthesis. We found no significant changes in Tph1 expression in the duodenum, colon, or bone of DMP1-caPTHR1 compared to its expression in WT littermates (Supplemental Fig. 1). Moreover, DMP1-Sost mice did not exhibit higher levels of Tph1 expression in either the intestine or bone, as would be expected if the low bone mass in these mice was due to increased serotonin (Supplemental Fig. 1). Consistent with our findings, Robling, Warman, and colleagues have not detected changes in Tph1 in the intestine of the low-bone-mass LRP5^−/−^ mouse or in the high-bone-mass LRP5 G171V knock-in mouse.(41) Taken together, these findings are inconsistent with the involvement of gut-derived serotonin in the bone formation changes exhibited by DMP1-caPTHR1 mice with or without LRP5 deletion or *Sost* overexpression, and they support the role of osteocyte-mediated regulation of the Wnt pathway on bone mass.

The higher periosteal BFR and cortical thickness exhibited by the DMP1-caPTHR1 mice contrast with the decreased periosteal BFR and thinner cortices found in mice in which the same active PTH receptor mutant is expressed under the control of the 2.3 fragment of the collagen 1a1 gene promoter (Col1a1-caPTHR1 mice), which is active not only in osteocytes but also in preosteoblasts and mature osteoblasts.(42) Direct comparison of micro-CT images of calvarial bones of 4-week-old mice confirmed that Col1a1-caPTHR1 mice exhibit decreased calvarial thickness, whereas DMP1-caPTHR1 mice exhibit increased calvarial thickness compared to WT mice of the same age (Supplemental Fig. 3). The divergent outcomes of activating the PTH receptor in different types of osteoblastic cells suggest that the positive effect on cortical bone formation induced by PTH signaling in osteocytes is counterbalanced by simultaneous activation of PTH signaling in osteoblast precursors or mature osteoblasts. This antagonistic effect might result from direct inhibition of periosteal osteoblast differentiation by PTH receptor activation, and it is consistent with the accumulation of immature osteoblasts with chronic elevation of PTH, such as in severe hyperparathyroidism(43) or in the Col1a1-caPTHR1 transgenic mice.(42,44) This phenomenon might also help explain the failure of PTH to increase bone formation in the periosteal surface in some conditions.(17–19)

The osteopenic phenotype of the DMP1-Sost transgenic mouse observed in vertebral cancellous bone is consistent with previous reports describing mice overexpressing human *Sost* under the control of the murine osteocalcin (OG-2) promoter(45) or under the control of the regulatory regions of the *Sost* gene.(46) However, cortical bone volume in the femoral mid-diaphysis was not decreased in our DMP1-Sost mice up to 2 months of age. This is in agreement with the milder decrease in femoral BMD compared to lumbar vertebra observed in OG2-Sost mice by Winkler et al.(45) Future studies will be required to determine whether the phenotype of our DMP1-Sost animals becomes more prominent with age in both the axial and appendicular skeleton, as observed in 5-month-old human *Sost* mice in the study by Loots et al.(46)

In spite of the different effect of the *Sost* transgene by itself on cancellous versus cortical bone, *Sost* overexpression equally abolished the increase in bone mass and volume induced by the DMP1-caPTHR1 transgene in both bone envelopes. These findings confirm that the anabolic effect of PTH receptor signaling in osteocytes requires *Sost*/sclerostin downregulation. Sost overexpression converted the bone gain exhibited by the DMP1-caPTHR1 mice into a bone loss in the DMP1-caPTHR1;DMP1-Sost mice. Thus, the double transgenic mice exhibit reduced total, femoral, or spinal BMD compared to animals expressing only the DMP1-Sost transgene and a persistent increase in osteoclasts in the face of absent Wnt signaling and reduced expression of Wnt target genes and osteoblast markers. Similar effects were observed in DMP1-caPTHR1 mice lacking LRP5. OPG expression was not different in DMP1-caPTHR1 mice compared to WT littermates, likely because of opposing effects of PTH receptor signaling on the OPG gene. Indeed, PTH inhibits OPG transcription by activating the cAMP-response element-binding protein (CREB),(47) and Wnt signaling stimulates OPG transcription(48,49) (Fig. 7*E*, *left panel*). Consistent with this, OPG expression was markedly decreased OPG expression was markedly decreased in DMP1-caPTHR1 mice lacking LRP5^−/−^ or overexpressing *Sost*, showing that when Wnt signaling is reduced, the cAMP-dependent inhibition of OPG expression prevails (Fig. 7*E*, *right panel*), resulting in a higher RANKL/OPG ratio. This is particularly noticeable in the double transgenic DMP1-caPTHR1;DMP1-Sost mice in which resorption is exacerbated, as evidenced by higher osteoclast number and calvarial marrow space compared to mice expressing only the caPTHR1 transgene. These observations, together with recent evidence showing that deletion of β catenin-dependent canonical Wnt signaling in osteocytes leads to reduced levels of OPG and increased resorption,(50) support a crucial role for osteocyte-derived OPG in bone resorption.

In closing, our study shows that activation of PTH receptor signaling in osteocytes dictates the recognized actions of PTH on formation and resorption in cortical bone and defines the role of the Wnt pathway. Acceleration of periosteal bone formation and apposition is due to downregulation of sclerostin, whereas the increase in osteoclast activity and intracortical remodeling is driven by osteocyte-dependent regulation of osteoclastogenic cytokines. Whether the mechanisms identified herein operate under conditions of endogenous elevation of PTH and/or administration of the hormone will require models of osteocyte-specific deletion of the PTH receptor. Our findings reveal that osteocytes are critical players in the regulation of cortical bone biology, opening new avenues to fracture prevention by targeting these most abundant bone cells.
