# Supplementary material for: PTH Receptor Signaling in Osteocytes Governs Periosteal Bone Formation and Intracortical Remodeling
Source: J Bone Miner Res. 2010 Dec 7;26(5):1035–46. doi: 10.1002/jbmr.304 (PMC3179307; doi:10.1002/jbmr.304)
Supplement: Supplementary file 1 [file jbmr0026-1035-SD1.ppt]

## Slide 1
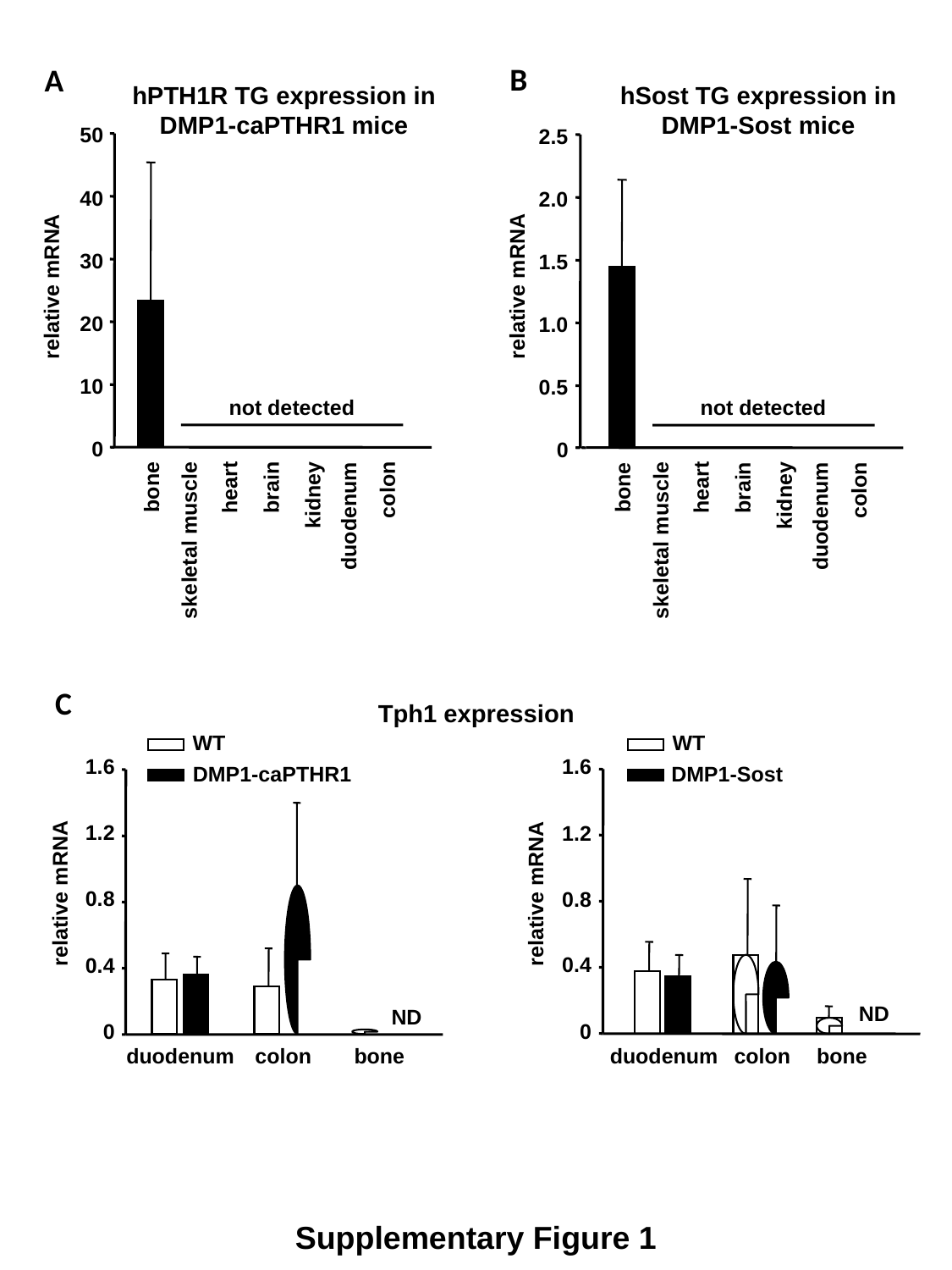

B
A
hPTH1R TG expression in
DMP1-caPTHR1 mice
hSost TG expression in
DMP1-Sost mice
50
2.5
40
2.0
30
1.5
relative mRNA
relative mRNA
20
1.0
10
0.5
not detected
not detected
0
0
bone
heart
bone
brain
heart
brain
colon
colon
kidney
kidney
duodenum
duodenum
skeletal muscle
skeletal muscle
C
Tph1 expression
WT
DMP1-caPTHR1
WT
DMP1-Sost
1.6
1.6
duodenum
colon
1.2
1.2
relative mRNA
relative mRNA
duodenum
colon
0.8
0.8
0.4
0.4
ND
bone
ND
bone
0
0
Supplementary Figure 1

## Slide 2
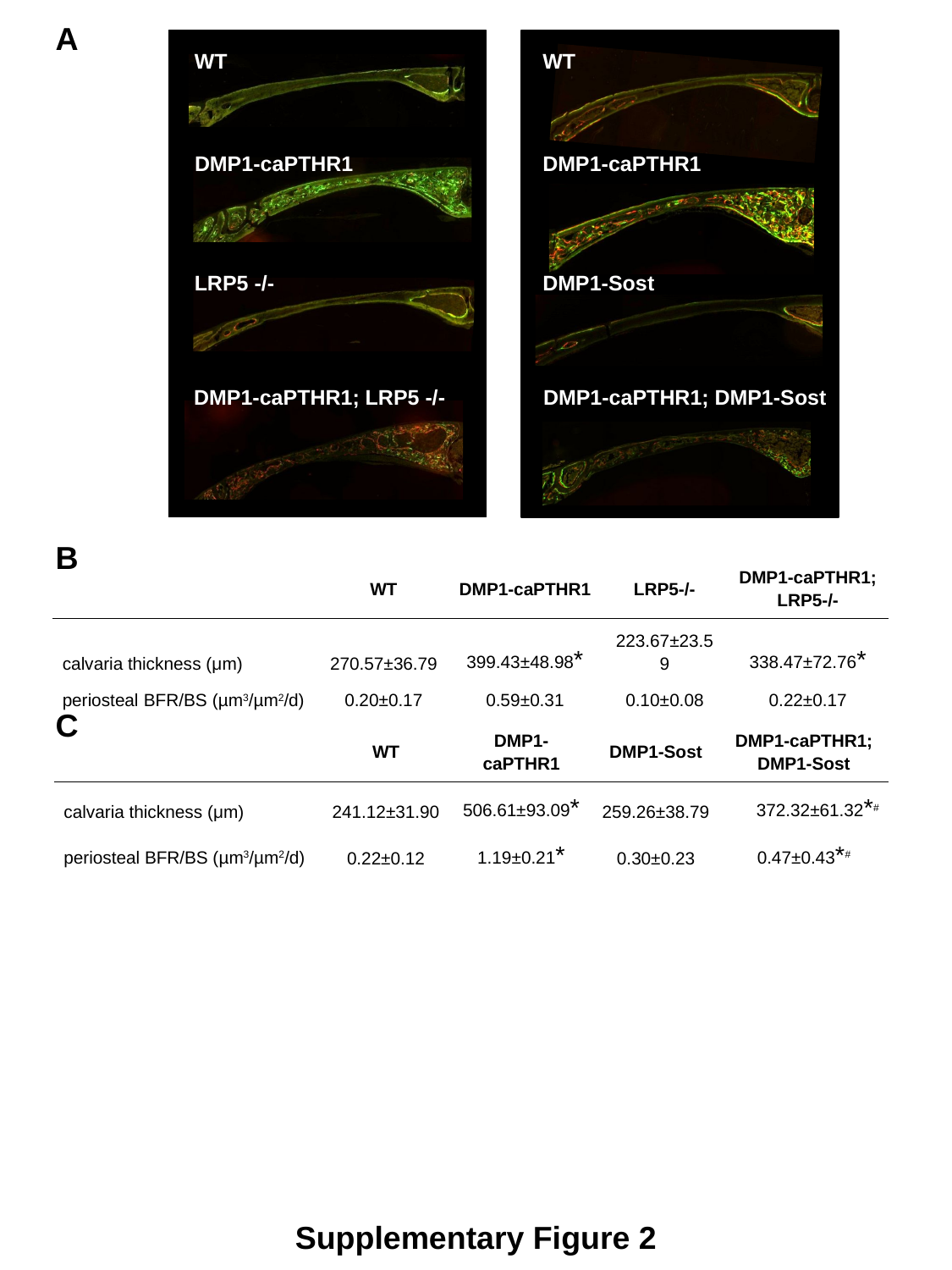

A
WT
WT
DMP1-caPTHR1
DMP1-caPTHR1
LRP5 -/-
DMP1-Sost
DMP1-caPTHR1; LRP5 -/-
DMP1-caPTHR1; DMP1-Sost
B
| | WT | DMP1-caPTHR1 | LRP5-/- | DMP1-caPTHR1; LRP5-/- |
| --- | --- | --- | --- | --- |
| calvaria thickness (μm) | 270.57±36.79 | 399.43±48.98\* | 223.67±23.59 | 338.47±72.76\* |
| periosteal BFR/BS (µm3/µm2/d) | 0.20±0.17 | 0.59±0.31 | 0.10±0.08 | 0.22±0.17 |
C
| | WT | DMP1-caPTHR1 | DMP1-Sost | DMP1-caPTHR1; DMP1-Sost |
| --- | --- | --- | --- | --- |
| calvaria thickness (μm) | 241.12±31.90 | 506.61±93.09\* | 259.26±38.79 | 372.32±61.32\*# |
| periosteal BFR/BS (µm3/µm2/d) | 0.22±0.12 | 1.19±0.21\* | 0.30±0.23 | 0.47±0.43\*# |
Supplementary Figure 2

## Slide 3
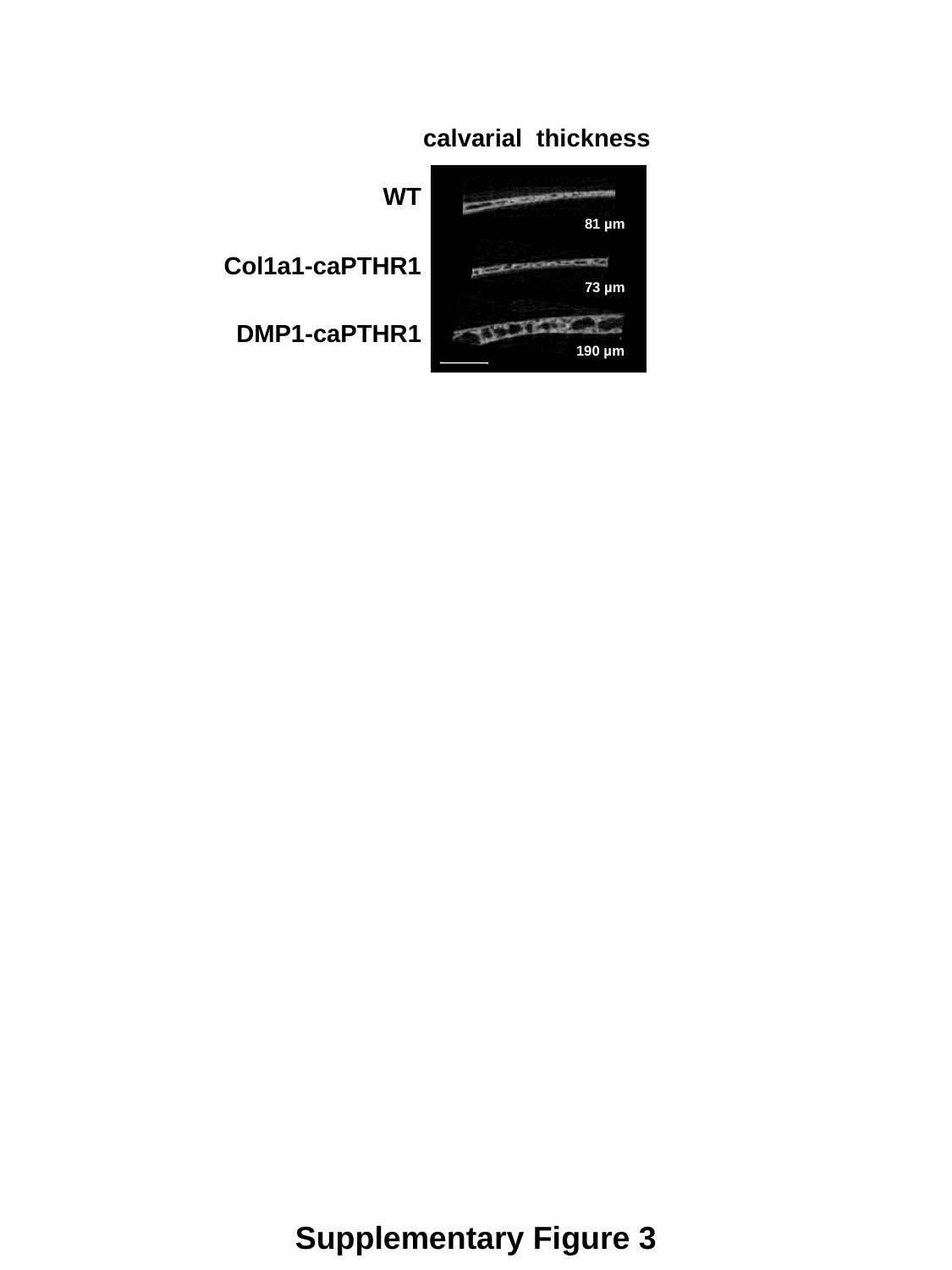

calvarial thickness
WT
81 µm
Col1a1-caPTHR1
73 µm
DMP1-caPTHR1
190 µm
Supplementary Figure 3
